# Unifying treatments for depression: an application of the Free Energy Principle

**DOI:** 10.3389/fpsyg.2015.00153

**Published:** 2015-02-20

**Authors:** Adam M. Chekroud

**Affiliations:** ^1^Department of Psychology, Yale UniversityNew Haven, CT, USA; ^2^Department of Neuroscience, Oxford UniversityOxford, UK

**Keywords:** major depressive disorder, predictive coding, free-energy principle, antidepressants, computational psychiatry, generative models, antidepressants efficacy

## Abstract

Major Depressive Disorder is a debilitating and increasingly prevalent psychiatric condition (Compton et al., 2006; Andersen et al., 2011). At present, its primary treatments are antidepressant medications and psychotherapy. Curiously, although the pharmacological effects of antidepressants manifest within hours, remission of clinical symptoms takes a number of weeks—if at all. Independently, support has grown for an idea—proposed as early as Helmholtz (von Helmholtz, 1924)—that the brain is a prediction machine, holding generative models^1^ for the purpose of inferring causes of sensory information (Dayan et al., 1995; Rao and Ballard, 1999; Knill and Pouget, 2004; Friston et al., 2006; Friston, 2010). If the brain does indeed represent a collection of beliefs about the causal structure of the world, then the depressed phenotype may emerge from a collection of depressive beliefs. These beliefs are modified gradually through successive combinations of expectations with observations. As a result, phenotypic remission ought to take some time as the brain's relevant statistical structures become less pessimistic.

## The free-energy principle

The free-energy principle has been proposed as a unifying framework that simultaneously links perception and action, and formalizes the roles of brain theories including attention, motor control, and perceptual learning (Friston et al., 2006; Friston, 2010; Clark, 2013). It is a mathematical description whereby the brain is a predictive device that builds statistical models of the world and then seeks to minimize “free energy,” an approximation of surprise. Free energy depends on a number of quantities, including the internal states of the brain, the external environment, and exchanges between the two (through action and perception). Mathematically, free energy is a statistical quantity that approximates the surprise in a sensory input, and it rests on two probability densities: a recognition density and a generative model (Friston et al., 2006; Friston, 2010; Clark, 2013).

The first component—the recognition density—is an approximate probability distribution of the causes of sensory data (Friston, 2010). This is quite a simple concept to apply: when a visual neuron fires in response to a horizontal bar in an area of the visual field, we could think that the stimulus caused the neural response. Equally, from the point of the neuron, we can consider its firing to reflect a probability that our sensation of a stimulus was caused by a horizontal bar in a particular area. The second component—the generative model—is a joint probability density between data and their causes from which samples can be drawn (Friston, 2010). In the present context, the generative model seeks to capture the statistical structure of its sensory environment by tracking the web of causes of that statistical structure. Crucially, the inferences we make about causes are not restricted to immediate sensory signals (e.g., “the switch caused light”), nor even time-varying/transitive inferences (e.g., “that bird is flying”) (Friston, 2010). The brain's model of the world also includes time-invariant regularities that afford structure to our world e.g., “gravity makes things fall,” but could equally be “I am in control of my own actions.” Having said this, it is important to note that Bayes rule and Bayesian brain theory do not guarantee veridical associations.

## Models in the brain?

A “belief” in the context of the free-energy principle is formalized as a probability distribution of an external state as internally represented by its sufficient statistics (Huys and Dayan, 2009; Friston, 2010; Mathys et al., 2011). For the purposes of this article, a “belief” is simplified as a prediction about the cause of an observation, given a particular circumstance and some previous experience. A “depressive belief” can then be considered as any consistent (negative) bias in these predictions, or vice versa.^2^ If our beliefs are to be useful, and reflect genuine associations rather than random co-occurrences we must consider the prior observations of all elements concerned (Fletcher and Frith, 2009). A simple thought experiment illustrates the concept of belief well: upon meeting a three-legged dog, one needs to recall all the previous times one encountered four-legged dogs to avoid the false prediction that dogs only have three legs.

Bayes' rule, a mathematical theorem, offers a mechanism for how beliefs should develop over time: updating as a function of past experiences (the prior), and the current experience (the likelihood) to produce a posterior belief or expectation. This interplay between likelihoods and priors may sound abstract, but it has the very practical implication that all our experiences depend on our knowledge of their predictability. The connection between the free-energy principle, predictive coding^3^ and the Bayesian brain rests on the fact that minimizing free energy corresponds to variational Bayesian inference. This may sound technical; however, it brings an important insight to the table: namely, all quantities involved in making predictions must jointly minimize surprise or free-energy. Notably, proposed quantities include synaptic activity (encoding beliefs about the current state of the world), synaptic efficacy (encoding regularities and causal structure) and synaptic gain (encoding the precision of beliefs) (Corlett et al., 2009, 2011; Adams et al., 2013). This three way split provides a natural framework to understand perceptual inference, learning, and the encoding of uncertainty, respectively. Crucially, to optimize any one set of these quantities one needs the optimal values of the others. The implicit circular dependency means that disruptions to inference, learning or the encoding of uncertainty will necessarily cause abnormalities in the other domains. Of particular importance here is the notion of precision. In predictive coding, precision corresponds to the (synaptic) gain applied to prediction errors and plays the role of a learning rate. We will return to this later when considering the link between neuromodulators, synaptic gain and their effects on perceptual inference and learning.

## Perception and belief: we see what we want to see

Exchanges between our brain's internal states and our external environment are bidirectional. That is, the brain draws its input through perception as it forms a model of the world, and then engages the external environment through action. It is this sampling of the environment that dictates our sensations, thus completing an action-perception cycle. Consider an intuitive example that occurs as we wander through our bedroom in complete darkness. We anticipate what we might touch in the world around us (expectations), and then feel around accordingly as we attempt to confirm these expectations (selective sampling). This process—whereby an agent selectively samples the sensory inputs that it expects—is known as active inference (Friston et al., 2009; Friston and Kiebel, 2009). In most real-life cases, there is already considerable contextual (i.e., prior) information in place when we encounter new information (Friston et al., 2006; Clark, 2013). There is, therefore, the potential for many prior expectations to be primed, alter the processing of incoming sensory information, and influence future environment sampling through action (Friston, 2010; Clark, 2013).

## When things go wrong

Predictions are only as good as the model that generates them. Disadvantages begin to creep into the system when beliefs become either inaccurate or inflexible (Fletcher and Frith, 2009; Ma, 2012). Recall that all of our experiences are influenced by our beliefs. Experiences that are in line with beliefs become predictable, strengthen the original belief and eliminate the need for the energy consuming processing of predictable sensations; because they have already been predicted and provide no “newsworthy” information. When an incorrect belief gains strength, it can result in one ignoring potentially informative experiences, or a range of other misattributions (Fletcher and Frith, 2009). The bidirectional belief-action relationship means that any inaccuracies in our model of the world might result in abnormal perception or action, and vice versa (Fletcher and Frith, 2009; Friston, 2010). Additionally, since the model must have a neural basis, correct predictions (in the form of some distributed neural network) could plausibly be disrupted by neurobiological changes.

## Changing the model: minimizing free-energy

Friston's original proposition offered two mechanisms for minimizing free-energy: through optimizing actions, and optimizing representations (Friston, 2010). In other words, we must either change the inputs to the model, or change the internal states. Returning to the example of walking through a dark room, there are two ways in which we might minimize surprise. One could sample differently (through action), e.g., turn on a light. Alternatively, one could change expectations (perceptual inference), e.g., entertain the alternative belief that you have woken up in a hotel room as opposed to your bedroom. It is critical to note here that both action and perception constitute an iterative cycle and depend upon each other. This contextualizes the three way dependencies between perceptual inference, learning and precision noted above: in other words, any changes in action rest upon changes in perception that—at some level—depend upon perceptual learning. Because perceptual learning proceeds at a much slower timescale than inference, our beliefs (which underlie action) do not change immediately; rather we successively combine past and current experience to optimize our generative model of the world. As such, rectifying a depressive model of the world (and thus the depressive phenotype) will be a gradual process. More specifically, this gradual process corresponds to the acquisition of generative models and involves the suppression of free-energy or prediction errors (over time) by changing connection strengths in the generative model. It is this process that one might consider to be the target of therapeutic interventions (e.g., by increasing learning rates—as is discussed later).

## Antidepressants: repairing representations?

Interesting parallels arise when considering depression from a free energy viewpoint. Anhedonia—a decreased interest in rewarding stimuli—is a cardinal symptom in the diagnosis of depression. Computational theories of reward-guided learning hold that future reward expectations depend heavily on the difference between actual and expected reward outcomes, i.e., prediction errors (Rescorla and Wagner, 1972; Sutton and Barto, 1998). Neurally, a close link has long been noted between prediction error signals and the firing of dopaminergic neurons during associative learning (Schultz et al., 1997). For instance, one recent study used optogenetic techniques to demonstrate a causal relationship between dopamine and anhedonia (Tye et al., 2013). Here, optogenetic silencing of midbrain (VTA) dopaminergic neurons was shown to induce a lack of sucrose preference (a homolog of anhedonia) in mice, while optogenetic stimulation of the same neurons relieved anhedonia (Hayes, 2013; Tye et al., 2013). However, while animal models suggest that phasic prediction error signaling is impaired in anhedonia, a recent behavioral meta-analysis of human data suggests otherwise (Huys et al., 2013).

Computationally, anhedonia can arise through either a primary insensitivity to reward, or disrupted ability to learning about rewards (Huys et al., 2013). Huys et al. (2013) directly contrasted these alternatives, conducting a model-based Bayesian meta-analysis of six datasets where depressed patients completed a probabilistic reward learning experiment. They found that reward sensitivity^4^ —but not learning—was impaired in MDD patients, but a dopamine agonist pramipexole showed the opposite pattern (Huys et al., 2013). Serotonin, on the other hand, is understood to selectively modulate behavioral and neural representations of reward value (Seymour et al., 2012). Specifically, it has been shown through acute depletion of a serotonin precursor (tryptophan) that serotonin depletion leads to impaired reward sensitivity in humans (Seymour et al., 2012). Other model-based differences in reward processing between depressed patients and controls have also been shown: depressed patients have blunted prediction error signals compared to healthy controls (Kumar et al., 2008; Gradin et al., 2011), and fail to adjust reaction times (e.g., post-error slowing) in the same way as control participants (Steele et al., 2007). As a brief but important note, either deficit would lead to inaccuracies in our recognition model: no longer faithfully reflecting reward causalities in our interactions with the world. Here we see important examples of how neuromodulation (serotonin and dopamine) can adversely affect beliefs about precision (sensitivity or gain and learning rates) to produce suboptimal inference and learning, respectively.

Seemingly abstract differences between computational quantities may carry important implications for the treatment of depression. Disruptions to neural representations of either reward sensitivity or reward learning would introduce inaccuracies to our generative model of the world. Indeed, a distorted mapping between actions and rewards could conceivably explain a number of depressive symptoms, particularly the feelings of hopelessness, distorted appetite, and anhedonia/decreased interest in pleasurable stimuli. However, the precise mechanism by which this occurs is critical to treatment strategies: while either impairment might lead to similar behavioral deficits, exactly which impairment a patient has carries implications for their treatment. At present, serotonin-targeting treatments are the first-line antidepressant method, but do not seem to alleviate depressive symptoms in many patients (Harmer and Cowen, 2013). After failed SSRI treatment, dopamine-targeting treatments can be attempted (Rush et al., 2006; Trivedi et al., 2006). It is entirely plausible that depressive symptoms, which broadly result from impaired reward processing, might stem from either, or both, impairments to serotonergic reward sensitivity and dopaminergic reward learning.

Perhaps, therefore, a reinforcement learning experiment might have predictive value over which treatment will be effective. If model-based analyses show a patient has a learning rate impairment, they may be more suited to dopaminergic treatment. If a patient has impaired reward sensitivity, perhaps serotonergic interventions ought to work. Considering behaviorally-dissociable recognition density distortions offers an interesting re-appraisal of inconsistent antidepressant success, with potential therapeutic implications. In fact, there is no reason to limit our investigation to these parameters. In one recent example (Diaconescu et al., 2014), sophisticated computational modeling was applied to a social learning task (based on Behrens et al., 2008) to investigate the mechanisms by which we infer the intentions of others. Such analyses characterize both social and non-social aspects of learning behavior extensively, and would enable researchers to consider potential abnormalities in MDD in a rich fashion. This kind of computational psychiatric approach is becoming increasingly popular, and has enjoyed recent success across a range of disorders (Montague et al., 2012; Corlett and Fletcher, 2014; Stephan and Mathys, 2014), including psychosis (Corlett et al., 2009, 2011), borderline personality disorder (Fineberg et al., 2014), schizophrenia (Fletcher and Frith, 2009), and delusions (Moutoussis et al., 2011).

## Rewiring beliefs

Since beliefs must be stored in the brain, using antidepressants to correct aberrant models of the world ought also to require some neurophysiological restructuring. This is in line with extant explanations for the delay in antidepressant efficacy. One early hypothesis for the delay in clinical effect of SSRIs argued that the desensitization of serotonin autoreceptors on serotonergic bodies and terminals is required before SSRIs can fully increase serotonin nervous transmission (Blier and de Montigny, 1994). In line with this suggestion, clinical trials combining SSRIs with serotonin autoreceptor antagonists have shown a faster and enhanced antidepressant effect (Whale et al., 2010). More recent alternative suggestions concerned the effects of SSRIs on neurotrophins and cellular processes generating new neurons and synapses. Animal models of depression highlight decreased BDNF production, neurogenesis, and synaptic plasticity: effects that are reversed by repeated administration of SSRIs (Santarelli et al., 2003; Castrén, 2005; Castrén and Rantamäki, 2010). Although interesting attempts have been made to apply the free-energy principle to monoaminergic (dopamine) transmission and inference (Friston et al., 2012), the present essay makes no prescriptions as to what specific neurobiological changes would happen as models become “less depressed.”

## Psychotherapy: breaking the action-perception cycle?

Correcting representations (the perceptual side to free-energy) might be one way of treating a depressive model of the world, but it is not the only way. Earlier I described the notion of active inference, whereby an agent selectively samples the environment in line with its model of the world, using the intuitive example of wandering in the dark (Friston, 2010). Another way in which we can influence the model is by changing its inputs, that is, optimizing actions to sample the environment differently. For instance, it may be that interactions with certain people or objects are further enhancing depressive symptoms, and/or (conversely) that a lack of positive actions is having a similar effect, reminiscent of learned helplessness models of depression. There is evidence in line with this; several studies have noted depressed patients spend significantly longer looking at negative stimuli (Matthews and Antes, 1992; Eizenman et al., 2003; Caseras et al., 2007; Seth, 2013)—perhaps this excessive negative sampling is skewing the inputs to our models. But this notion also extends beyond an individual's physical actions; excessively sampling negative causal relationships might also distort an agent's model of the world. Indeed, this idea of active sampling concurs with recent theoretical work linking interoceptive inference and emotion, where emotion is held to emerge from cognitive appraisals of physiological states (Seth, 2013). One recent computational paper attempted to model emotional valence as the second time-derivative of free-energy, where emotional valence regulates the learning rate of the causes of sensory inputs (Joffily and Coricelli, 2013). In more plain terms: “when sensations increasingly violate the agent's expectations, [emotional] valence is negative and increases the learning rate. Conversely, when sensations increasingly fulfill the agent's expectations, [emotional] valence is positive and decreases the learning rate” (Joffily and Coricelli, 2013). Put simply, active inference requires us to sample the world in accordance with our expectations. If expectations imply the world is a rather hostile place, then it will be sampled as such. There are clear analogies with learned helplessness models of depression here. Interestingly, learned helplessness can be (Bayes) optimal, if the world is indeed persistently hostile and has a low volatility.

Note again the crucial role of the learning rate in facilitating the (re)learning of a generative model. Under predictive coding, an implementation of the free-energy principle, the learning rate increases with the expected volatility of environmental contingencies, but volatility is only one factor influencing it (Mathys et al., 2011, 2014). Behaviorally, it has been shown that healthy human subjects assess volatility in an optimal manner—that is, increase their learning rate when the environment is more volatile (Behrens et al., 2007). In this study, the authors demonstrated that the optimal estimate of environmental volatility was reflected in the fMRI signal in the anterior cingulate cortex (ACC), and variations in this signal predicted between-subject variations in learning rate. Although no study has specifically investigated the ability of depressed patients to optimally update learning rate according to their environment, one study showed that controls—but not patients—significantly activated the ACC when given negative feedback during a gambling task (Gradin et al., 2011). Of course, this itself does not mean that ACC activity significantly differed between controls and patients (Gelman and Stern, 2006).

Nonetheless, it seems optimizing actions in order to change a model of the world is reflected in psychotherapy approaches. APA (2010)^5^ guidelines for depression psychotherapy include helping people “gradually incorporate enjoyable, fulfilling activities back into their lives,” and “improve patterns of interacting with other people that contribute to their depression,” both of which would constitute optimisation of actions under the free-energy framework. Essentially, breaking any actions or sampling mechanisms that further a depressive model of the world appears to be a recommendation that the free-energy framework makes, that psychotherapy treatments have already taken.

## Free-energy: a holistic approach?

**Figure 1:**
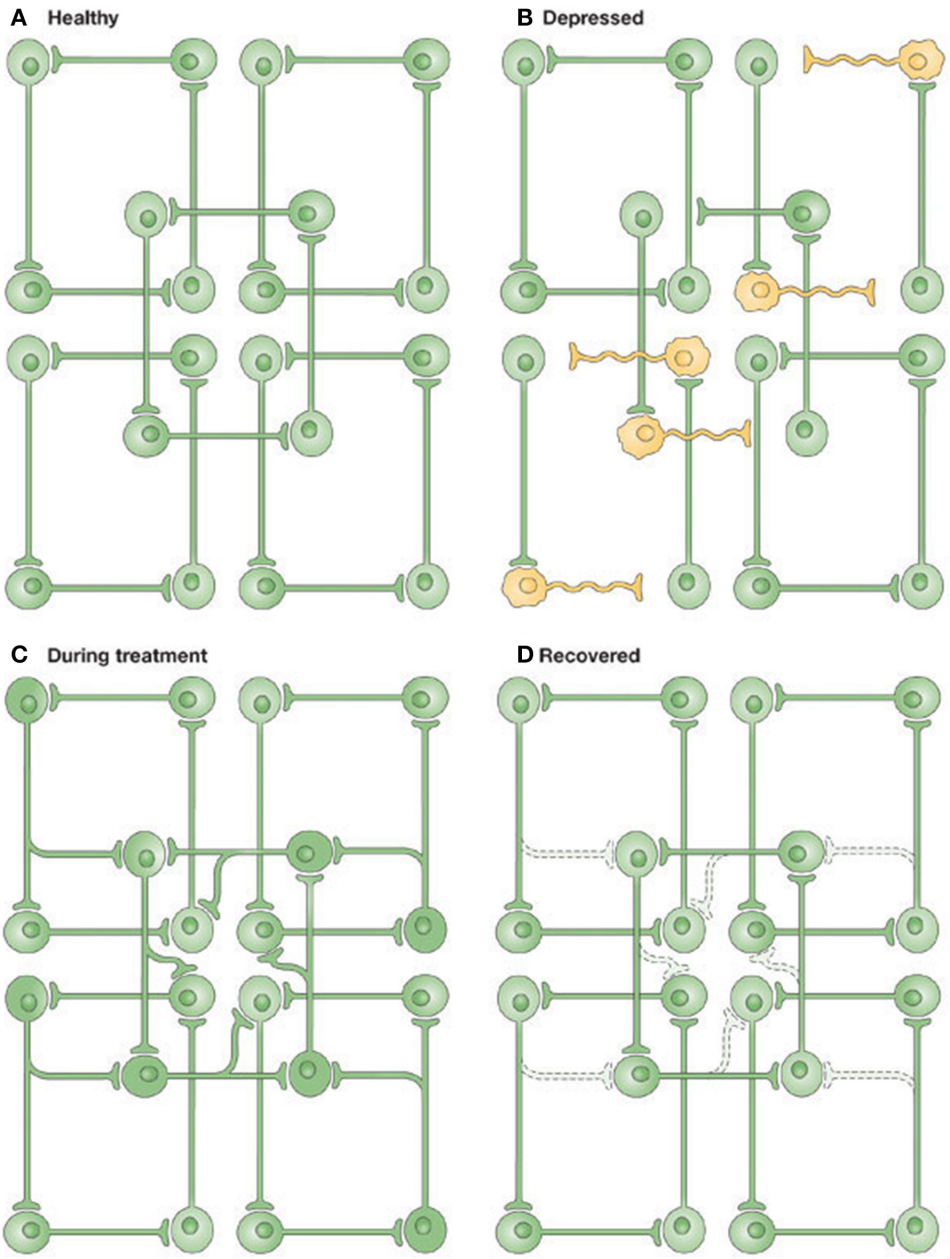
**Illustrating the network hypothesis of Depression**. **(A)** In the healthy brain, information is distributed amongst partially overlapping brain networks. **(B)** In Depression, some information processing is altered. **(C)** Antidepressant treatments enhance connectivity in neural networks. **(D)** Activity-dependent synaptic pruning stabilizes the network. Figure and caption reproduced with permission from Castrén (2005).

It is worth briefly setting this approach in the context of other accounts of depression. It is true that we already have elegant emotional/cognitive accounts of depression (Harmer and Cowen, 2013), and there are many putative biological explanations of disruption and restoration at cellular (Castrén and Rantamäki, 2010) and molecular (Berman et al.) levels. However, pharmacological level explanations often lose sight of the multidimensional nature of the depressive phenotype; and emotional or high-level explanations are difficult to relate directly back to the neurobiology. In fact, a predictive coding approach resembles a previous information-processing level approach, illustrated in Figure 1, known as the “network hypothesis of depression” (Nestler et al., 2002; Castrén, 2005). At a minimum, this is encouraging: it suggests that the free-energy framework is largely consistent with theories of depression at multiple levels, and offers a plausible alternative that also unifies global brain theories in biological and physical sciences. In future, it may offer an opportunity for researchers to directly transition between depression's many levels of research in a principled, model-based fashion (Montague et al., 2012; Friston et al., 2014; Stephan and Mathys, 2014). For example, there may be different underlying problems in MDD, with different behavioral ways of testing, with specific therapeutic implications.

It is also interesting to relate the free-energy approach to the literature on Depressive Realism, a claim that depressed people are sometimes better at evaluating instrumentality than non-depressed people (Alloy and Abramson, 1979; Alloy et al.). The claim appears robust, if small: a recent meta-analysis of 75 studies indicated a small overall depressive realism effect, although both depressed and non-depressed individuals showed a substantial “optimism bias” (Moore and Fresco, 2012). Some compelling model-driven research suggests the effect may be driven by contextual processing differences, rather than depressed individuals having consistently low expectation of control (Msetfi et al., 2005). This is partially supported by one recent pharmacological study showing that amongst a group of 15 non-depressed participants, acute tryptophan depletion improved contingency judgments for participants with particularly low scores on the Beck Depression Inventory (BDI < 6; Chase et al., 2011). In a free-energy view, “control” in the clinical psychological context corresponds to outcome entropy, and it directly influences an individual's belief about what kinds of outcome distributions are likely. “Maladaptive” priors or generalization tendencies could equally result in differences in perceived control, although “maladaptive” here requires some clarification. Since both depressed and non-depressed individuals typically show an optimism bias, “maladaptive” is simply with reference to non-depressed individuals, rather than a comment on optimality. Although a detailed analysis of entropy and perceived control is beyond the scope of the current article, Huys and Dayan (2009) offer an excellent mathematical treatment of behavioral control from a Bayesian perspective.

The free-energy approach detailed in this review is not, however, an exhaustive account of depression. Symptoms of low mood and anhedonia may be cardinal symptoms in MDD but they are not the only ones: the accompanying loss of appetite, sleep disturbance, diurnal fluctuation, low energy and somatic symptoms are a key part of the illness. Furthermore, these additional symptoms can sometimes be the ones that are slowest to resolve. It is possible that wider symptoms may emerge as a behavioral consequence of a distorted generative model: for instance, if food rewards are no longer subjectively rewarding then loss of appetite or motivation to eat is understandable, if not predictable. In addition, although this review focused on the most common treatments for depression—monoaminergic antidepressants and psychotherapy—there is now preliminary evidence that intravenous administration of ketamine and other glutamatergic drugs can have remarkably quick—but transient—antidepressant effects in unipolar and bipolar depression (aan het Rot et al., 2010; Aan Het Rot et al., 2012; McGirr et al., 2014). The speed of ketamine's antidepressant efficacy here may appear problematic for a free-energy interpretation at first glance. However, few treatments in psychiatry or medicine are effective after a single dose, and ketamine is no exception: patients often return to the depressed state without a course of treatment over a number of weeks (aan het Rot et al., 2010; McGirr et al., 2014). From a free-energy perspective, ketamine can be considered a faster vehicle for repairing representations, but one that nonetheless takes some time to repair the generative model. In addition, from a neurobiological perspective, ketamine's acute and sustained antidepressant effects have been hypothesized to depend on synaptogenesis (Li et al., 2010), in reminiscent fashion to monoaminergic antidepressants. Further insight comes from Bayesian treatments of psychosis using ketamine as a model (Corlett et al., 2009, 2011). Here, distinct influences have been proposed for ketamine in the short and long term. In the short term, it is thought that ketamine briefly disturbs cortical inference by blocking NMDA receptors, and impairing the specification of top-down prior expectancies (Corlett et al., 2011). With chronic ketamine use, however, there is a compensatory increase in the number and function of NMDA receptors; longer-lasting changes that can give way to a delusional phenotype and depressed mood rather than remission from depression (Morgan et al., 2010; Corlett et al., 2011).

## Conclusion

Under the free-energy principle the brain is an active prediction engine that seeks to establish a model of the causal structure of our environment, and minimize long-term surprise. The brain makes inferences about causal relationships at many levels of abstraction, and there is growing neural evidence in line with this theory. If the brain does indeed represent a collection of beliefs about the causal structure of the world, then the depressed phenotype emerges from a collection of depressive beliefs. The two mechanisms by which free-energy is minimized (and perhaps, how agents survive) are by optimizing actions, and optimizing representations. The two are markedly reminiscent of depression's two main therapies: psychotherapy and antidepressants, respectively. Distorted representations of the world might stem from distortions in reward representation, and correcting these through monoaminergic interventions might be a solution to anhedonia symptoms in particular. Similarly, a distorted sampling mechanism may exacerbate depressed mood, and require psychotherapies in an attempt to break the spiral of self-defeating actions. Either way, solutions ought not to be immediate: beliefs are changed gradually through successive combinations of past experiences and current observations. Irrespective of the formal insights into putative pathophysiology in depression, it may be the case that the holistic (theoretical) framework on offer here may be useful in cognitive behavior therapy. In other words, it may provide a rationale for the conjoint use of psychotherapeutic and pharmacological approaches that could be useful for both the therapist and patient alike. One thing is clear: depression is a multi-faceted illness in which disruptions to beliefs, emotions, perception and action are intertwined. Perhaps, therefore, our approach must intertwine beliefs, emotions, perceptions and actions accordingly.

### Conflict of interest statement

The author declares that the research was conducted in the absence of any commercial or financial relationships that could be construed as a potential conflict of interest.

## Footnotes

^2^Interested readers are encouraged to read an excellent formal treatment of model inversion by FitzGerald et al. (2014), which explores the implications of approximate Bayesian inference on behavior.

^3^Predictive coding refers to a class of theories in which the brain is held to continually generate models of the world based on context and information from memory to predict sensory input (Rao and Ballard, 1999; Friston and Kiebel, 2009; Clark, 2013).

^4^It may be important to clarify that while the Huys paper demonstrated impaired reward sensitivity for *wanting* in MDD patients, it seems that *liking* rewards remains intact in MDD (Dichter et al., 2010).

^5^Available online at: http://psychiatryonline.org/pb/assets/raw/sitewide/practice_guidelines/guidelines/mdd.pdf
